# Draft Genome Sequences of Three *Bacillus* Species Isolated from Biofouled Reverse-Osmosis Membranes

**DOI:** 10.1128/MRA.00413-20

**Published:** 2020-07-09

**Authors:** Zahid ur Rehman, Muhammad Ali Iftikhar, TorOve Leiknes

**Affiliations:** a Water Desalination and Reuse Center, King Abdullah University of Science and Technology, Thuwal, Kingdom of Saudi Arabia; b Department of Ophthalmology, Luton and Dunstable University Hospital NHS Foundation Trust, Luton, United Kingdom; University of Southern California

## Abstract

Here, we present the draft genome sequences of three bacteria belonging to the genus *Bacillus* which were isolated from biofouled reverse-osmosis (RO) membranes harvested from a full-scale desalination plant. The sizes of the assembled genomes for RO1, RO2, and RO3 were 4.22 Mb, 4.15 Mb, and 4.23 Mb, respectively.

## ANNOUNCEMENT

The bacteria belonging to the genus *Bacillus* have expansive physiology that allows them to colonize diverse environments such as soil, water, air, and lake sediments, as well as extreme environments, including salt marshes, hot springs, and acid water (1). The bacilli in seawater can initiate biofouling of reverse-osmosis (RO) membranes (2), which leads to increased maintenance and water treatment costs. In addition, bacilli have been shown to produce xanthine oxidase, a free radical-generating enzyme that can be implemented in biological control of fouling (3). Therefore, these bacteria provide a valuable resource for studies on the formation of biofouling and its control.

A 7-year-old biofouled RO membrane module was obtained from a seawater desalination plant located on the coast of the Red Sea in Saudi Arabia (22.299815N, 39.116812E). A 1-cm^2^ membrane piece was cut, mixed with 10 ml of 1× phosphate-buffered saline (PBS), and vortex mixed. After mixing, 100 μl of PBS was plated onto freshly prepared marine agar plates and incubated at 30°C for 72 h. Three phenotypically distinct (based on color) colonies were selected and streaked onto fresh marine agar plates. This procedure was repeated three times to obtain pure cultures of the isolates.

For DNA extraction, all of the isolates were grown in 20 ml of marine broth for 48 h, with shaking at 120 rpm. The cell culture was centrifuged at 6,000 × *g* for 10 min, and the resulting cell pellet was used for DNA extraction using the DNeasy PowerWater kit (Qiagen, Germany). The NEBNext Ultra II DNA library preparation kit (New England Biolabs, USA) was used to prepare the sequencing library according to the manufacturer’s instructions. The library preparation and sequencing were performed by DNASense (Aalborg, Denmark). Paired-end sequencing (2 × 301 bp) of the samples was performed on the MiSeq platform (Illumina, USA) using the MiSeq reagent kit v. 3 (600 cycles).

The bioinformatic processing of the sequence reads was performed using default parameters for all software unless otherwise specified. The sequencing reads were trimmed using Cutadapt v. 1.16 (4) and assembled using MEGAHIT v. 1.1.3 (5). The genomic features of all of the strains are given in Table 1. The genomes were annotated using the NCBI Prokaryotic Genome Annotation Pipeline (PGAP) v. 4.11 and Prokka v. 1.14-dev (6, 7). The numbers of protein-coding genes, rRNAs, and tRNAs detected for RO1, RO2, and RO3 are given in Table 1.

**TABLE 1:** Genomic features of RO isolates

Genomic feature	Data for strain:		
	RO1	RO2	RO3
Assembly length (bp)	4,224,738	4,154,828	4,223,915
No. of contigs	92	57	34
No. of reads	1,610,647	2,107,415	1,860,114
GC content (%)	40	40	43
Contig *N*_50_ (bp)	92,363	118,722	226,369
Completeness (%)	98.85	98.56	98.56
Coverage (×)	217	268	238
No. of protein-coding genes	4,049	4,059	4,250
No. of tRNAs	65	38	79
No. of complete rRNAs	14	10	6

The taxonomic assignment of the genome by GTDB-Tk v. 1. 0.2 (8) showed that RO1 and RO2 are related to *Bacillus* sp. CHD6a (average nucleotide identity [ANI] values of 91% and 92.2%, respectively), while RO3 is related to Bacillus aquimaris (ANI value of 83.1%). The ANI values were below the species demarcation limit (ANI values of ≥95%) (9, 10), which suggests that these isolates may represent novel species of the genus *Bacillus* and thus may require new species names.

### Data availability.

The genome sequences reported in this article were deposited in DDBJ/ENA/GenBank under BioProject number PRJNA616073 and accession numbers JAAZWB000000000 (RO1), JAAXCU000000000 (RO2), and JAAXCV000000000 (RO3). Raw reads were deposited in the Sequence Read Archive (SRA) under accession numbers SRR11849547 (RO1), SRR11849546 (RO2), and SRR11849545 (RO3). The genomic versions described in this paper are versions JAAZWB010000000, JAAXCU010000000, and JAAXCV010000000.
